# Alliance with the School Personnel Is Crucial for the Management of Food Allergy and Anaphylaxis in School Children

**DOI:** 10.3390/foods10092083

**Published:** 2021-09-03

**Authors:** Dimitris Efthymiou, Persefoni Panayi, Gavriela Feketea, Constantinos Pitsios, Ioana Adriana Muntean, Emilia Vassilopoulou

**Affiliations:** 1 2nd Department of Psychiatry, Division of Neurosciences, Medical School, Aristotle University Thessaloniki, 54124 Thessaloniki, Greece; dimitrisefthy@gmail.com; 2 Allergy Outpatient Clinic, Medical School, University of Cyprus, P.O. Box 2537, 1678 Nicosia, Cyprus; panayi.persefoni@ucy.ac.cy (P.P.); pitsios.contantinos@ucy.ac.cy (C.P.); 3 PhD School, “Iuliu Hatieganu” University of Medicine and Pharmacy, 400337 Cluj-Napoca, Romania; g.feketea@karamandaneio.gov.gr; 4 Pediatric Allergy Outpatient Clinic, Department of Pediatrics, “Karamandaneio” Children Hospital, 26331 Patras, Greece; 5 Department of Allergology and Immunology, Institute of Gastroenterology and Hepatology “O Fodor”, “Iuliu Hatieganu” University of Medicine and Pharmacy, 400162 Cluj-Napoca, Romania; 6 Department of Nutritional Sciences and Dietetics, International Hellenic University, 57400 Thessaloniki, Greece; vassilopoulouemilia@gmail.com

**Keywords:** food allergy, anaphylaxis, school, school nurses, epinephrine

## Abstract

Background: School nurses play an important role in coping with food allergy (FA) in schoolchildren, but in schools with no school nurse, the school personnel should be prepared to manage health emergencies. This study aimed to evaluate allergy management competences in primary schools in Cyprus. Methods: The study was conducted September 2016 to May 2017 in 11/13 primary schools, selected by stratified random sampling. Information was collected from a principal/designated teacher using a questionnaire from the EuroPrevall Project, adapted for Cypriot teachers. Results: An average of six children with FA per school was reported in the preceding three years. Protocols for the management of chronic diseases, including allergies, were in place in 8/11 schools. Regarding recognition of FA, 8/11 respondents knew some of the signs and symptoms. In an allergic emergency, 9/11 would call the child’s parent/caregiver first and 2/11 emergency medical support. Epinephrine auto-injector (EIA) was reported by 2/11 respondents to be available in the school, but only one reported training in its use. Conclusions: The preparedness of primary schools in Cyprus did not meet safety standards regarding the preparedness of school personnel to cope with an allergic reaction in children with FA, including the use of EIA.

## 1. Introduction

Food allergy (FA) is a common pediatric emergency and constitutes a significant concern for the personnel of preschool facilities and schools. Its prevalence varies in different parts of the world, and in Europe it affects about 6% of primary school children [1,2,3]. Even when children with FA are trained to avoid the offending foods, accidental FA reactions may occur, about 18% of these at school [4,5,6]. Cow’s milk is the most common allergen causing reactions in preschool children, and peanuts in schoolchildren, but culprit allergens are related to the eating habits affected by the varied diets consumed in different countries [6,7,8]. FA and anaphylaxis related policy in schools, and the legislation concerning treatment planning, both vary considerably from country to country, and even from school to school in the same country [9]. Even though strict avoidance of the offending food is the main form of management for FA, an individualized emergency plan, including use of the epinephrine auto-injector (EAI), is necessary for the treatment of food anaphylaxis [10]. Emergency plans are usually put in place after a confirmed diagnosis of FA by allergists and pediatricians, both of whom can play an important role in collaboration with school personnel [11]. Proactive parents train their children to eat only home-provided food, and they communicate the diagnosis and the emergency plans to the children’s school nurses, teachers and/or principals. By minimizing the risk of anaphylaxis at school, a normal life can be offered to the child with FA, with no deprivation of any of the school activities [1].

Unfortunately, the current situation regarding the prevention and management of anaphylaxis by school personnel is not ideal. Even though school nurses can have an important role in promoting the health and well-being of school-aged children, they are not employed in the state schools in most countries [12]. The school personnel are usually not sufficiently trained to recognize allergic reactions and to help children with FA [13]. The willingness of school personnel to cooperate, to be trained and to acquire confidence in handling children suffering from FA, and to cope with the legislation framework, are parameters that need to be cultivated. School personnel are important stakeholders, and they can and should be prepared to manage health emergencies in children [14]. We conducted the present study as part of the first epidemiological study on FA in Cypriot primary school children [2,3], with the purpose of evaluating the allergy management competences in primary schools in Cyprus, and to explore the knowledge and beliefs of schoolteachers about FA.

## 2. Materials and Methods

The study was conducted between September 2016 and May 2017 in 11 of the 13 primary schools invited to participate, with approximately 300 pupils attending each school. The selection of the schools was based on the diverse geographical location and number of local schools, in both urban and rural areas of Cyprus. Schools from the four larger cities (Nicosia, Limassol, Larnaca and Paphos), and from the provincial areas of both mountain and coastal villages of the island, were included by stratified random sampling, as previously described [2]. This investigation was conducted in parallel with a survey on food-hypersensitivity in the pupils attending the schools under question. The age range of the schoolchildren in the study schools was 5.5–12 years (mean 8.7 ± 1.7 years) [3]. The school principal of each school, or a schoolteacher designated by the school principal as the most appropriate person to provide the required information on the school preparedness for FA was assigned to participate in the study. The semi-structured questionnaire used in the EU-funded multidisciplinary Integrated Project EuroPrevall, previously translated and adapted for use in Greek populations, was used [15]. The study was approved by the Cyprus National Bioethics Committee (EEBK 2017.01.47) and the Department of Education of the Cyprus Ministry of Education, Culture, Sport and Youth.

The questionnaire (Table 1) is a comprehensive tool, consisting of 42 questions, mainly focusing on the awareness of the school personnel of FA reactions and the plans for the management of medical emergencies in the school where the principal or teacher is employed. Most of the questions are open-ended and they were addressed to the respondent as the authorized member of the school personnel. The respondents were assured of the anonymity of the research data, and the protection of personal details was ensured by the interviewer.

The data were analyzed with descriptive statistics using SPSS 22.

## 3. Results

The school principal was the designated interviewee in 8/11 schools, and a teacher was appointed to be interviewed as school representative in 3/11 schools. Each of the participating schools had an average of 25 teachers in the personnel.

**Figure 1:**
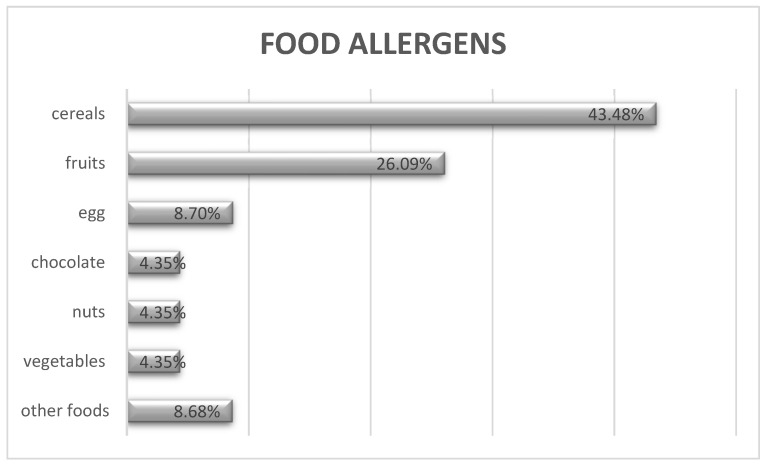
Food allergic reactions per food allergen, according to the school medical card, as reported by parents/carers (*N* = 68).

The majority (10/11) of the participating schools (90.9%) had been informed by parents, in the preceding three years, that one or more pupils had a medical history of FA. An average of six children with FA per school was estimated. Specifically, 68 cases of FA had been reported, but only 10 of these were reported during the last 3 years. The foods most commonly listed by the parents as causing the allergic reaction were (Figure 1): cereals including wheat, barley, rye, maize, rice and oats (43.48%), fruits including orange, strawberry, cherry and peach (26.09%), egg (8.7%), chocolate (4.35%), tree nuts (4.35%) and vegetables (4.35%). Protocols for the management of chronic diseases, such as allergies, were reported to be in place in 8/11 schools, and to be lacking in 3/11.

Regarding the ways used to identify the children with FA, 8/11 respondents replied that they were informed through the standard mandatory school child’s medical card, which is completed yearly by the parents/caregivers [16]. The additional use of medical identifications (IDs), such as medical alert bracelets worn by children, was reported in one school. A list of the children with FA, updated every year, recording the medical history and the culprit allergens, was reported to be kept in 2/11 schools. All members of the school personnel have access to this list.

**Figure 2:**
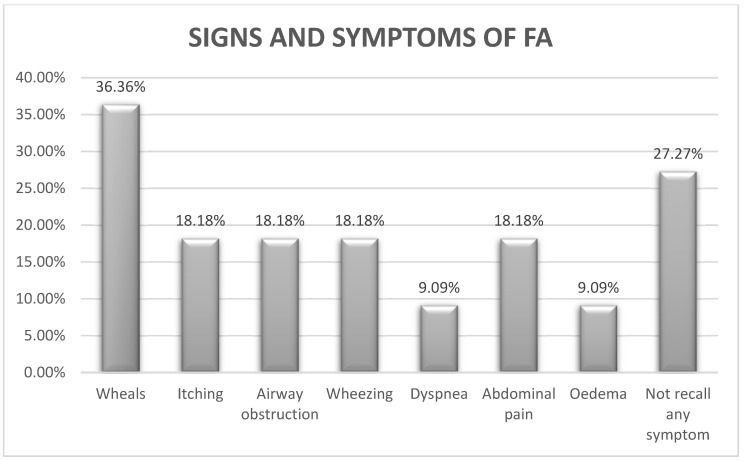
Different types of signs and symptoms of FA stated by teachers during an allergic reaction.

Regarding recognition of the signs and symptoms of FA, 8/11 respondents stated that they know some of the signs of FA. They mentioned seven types of signs and symptoms: wheals (4/11), itching (2/11), airway obstruction (2/11), wheezing (2/11), dyspnoea (1/11), abdominal pain (2/11) and oedema (1/11), but 3/11 could not recall any symptom. The personnel had received training relevant to allergies and allergic symptoms, and were prepared to manage an allergic reaction in a child, in only 2/11 schools (Figure 2).

In these two schools, relevant educational seminars had been provided for the whole school personnel, twice in the preceding three years, by an allergist and a dietitian. 

The respondents agreed that an emergency protocol, determined by the Cyprus Ministry of Health, for the provision of first aid by teachers, including the management of severe allergic reactions in the school environment [16] is vital. As a first step in the case of an allergic emergency, 9/11 respondents would first call the child’s parent/caregiver and 2/11 would first call for emergency medical support. EIA (Anapen) was reported to be available in the school by 2/11 respondents, but they doubted whether all the school staff knew of the availability of Anapen in the school, and only one stated that any of the personnel was trained to use EIA.

Directions issued by the Ministry of Health on how to treat FA and anaphylaxis [16], were reported in 8/11 schools, but such directions were absent from, or were not used in 3/11 schools. Only 1/11 school enforced rules restricting the sharing of cutlery, glass and foods, in order to prevent accidental allergic reactions. All the respondents reported that most members of the personnel ignore such rules and that, therefore, there is a risk of accidental exposure to food allergens. All the respondents, however, agreed that such a protocol would not affect the development of the personality and generosity of children but, on the contrary, it would raise their awareness of on how severe an allergic reaction can be.

In 3/11 schools, regular personnel training is provided by an allergist and a dietitian on food labeling, and the pupils also receive this type of training, integrated into their school program. In these three schools it was also reported that the rules of allergen labeling are followed strictly in their cafeterias, and that certain foods had been excluded, because of the absence of appropriate labeling, with the ingredients not clearly indicated according to the relevant legislation rules (Regulation EU No 1169/2011).

**Figure 3:**
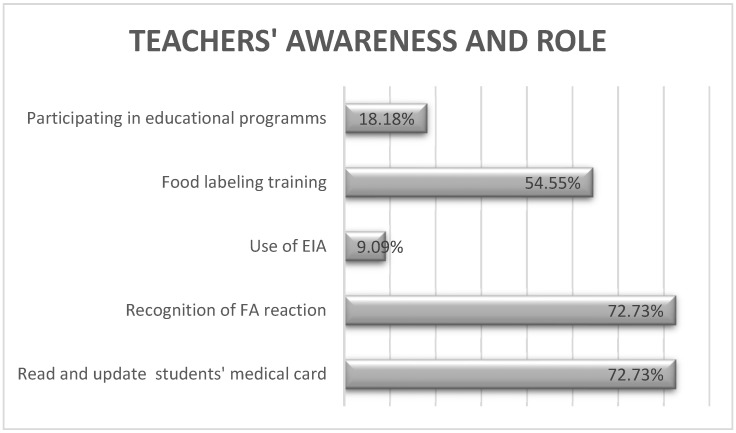
Percent of teachers that have different skills and knowledge about FA children in the school environment.

Of the respondents, 4/11 were greatly concerned about the possibility of food-induced allergic reactions at school, scoring > 70 on a 0–100 scale. The main reasons for their concern were their unfamiliarity with the symptoms and severity of food anaphylaxis, and their lack of confidence in managing such a reaction in the school environment. Almost all stated the need of an educational program for school staff and parents, aimed at raising awareness about FA reactions and competence in coping with them, and 5/11 considered the study interview a motivation to learn more about this medical issue. The different skills and knowledge of teachers regarding FA children is depicted in Figure 3.

## 4. Discussion

Management of FA at school constitutes a challenge to the personnel. It includes the prompt recognition of the signs and symptoms of an allergic reaction, and rapid application of the appropriate treatment, following a specified emergency plan and using the prescribed emergency set [17]. In one relevant study performed in six schools in Houston, 59% of teachers failed to identify an allergic reaction or to follow an emergency plan [5]. The prompt recognition of signs of anaphylaxis and the implementation of the prescribed management plans are crucial. Most of the respondents in the participating Cypriot primary schools reported knowledge of the symptoms of food allergies. In an Icelandic study on children in preschool, only 55% of preschool facilities with children with severe allergy reported that all of their staff members knew the symptoms related to anaphylaxis [18].

The literature supports the high incidence (16–18%) of FA reactions in the school environment [6,19]. The preparedness of school personnel for intervention, including the use of EIA, is indispensable in the case of an anaphylactic reaction in a child at school. Despite the guidelines provided for the provision of first aid, including for anaphylaxis, by teachers [16], a gap in the awareness of the use of EIA in Cypriot primary schools emerged from our study, as has been reported in other studies on the awareness of relevant school procedures on the part of the school personnel and parents [20].

The Cypriot legislative framework states that providing first aid to children with chronic diseases that may present an acute exacerbation, is a moral duty of teaching personnel and a principle of school rules. Teachers should be well informed on the health problems of pupils and instructed on the steps to be followed in relevant health emergencies. Medication may be administered by a trained teacher on the physician’s instruction or with the written consent of the parent (Ministry of Health 21.1.07.2). In spite of the legislation, we noted lack of access to appropriate medical treatment (i.e., EIA) and/or education in its administration, and absence of standard protocols on how to react in the case of an emergency. The respondents in our survey reported that in the event of an allergic reaction they would contact the parents first, rather than the medical emergency services.

These gaps in the implementation of guidelines at school are common. Parents were described as the primary drivers of FA guidelines implementation in an Australian study [21]. In a survey in Japan, school staff members reported attending courses about FA, but on being questioned before and after the course, only 34.5% of school teachers and principals knew the indications for using adrenaline in children [22]. An Italian study reported that while the majority of school teachers and principals (65.4%) knew that “adrenaline” is the best medication for anaphylaxis, only 34.5% knew the indications for using it [23].

Teachers need to know that, in a child with FA, a reaction is probable with even minimal exposure, so that sharing food with a child who has FA [17], or letting a child who is allergic to egg use egg-containing finger paints, can be extremely dangerous [24]. Labelling of processed food is crucial, since a hidden or misinterpreted ingredient can trigger an anaphylactic reaction, which, in combination with the deficiency of knowledge of EIA of the school personnel can be life-threatening [25]. The majority of the respondents in our study reported lack of a school policy on avoiding the sharing of cutlery, glasses and foods, but also lack of training on food labelling.

Educational seminars for school personnel on FA, including first aid courses, part of which is the treatment of allergic emergencies, are of utmost importance [15]. Such intervention increases the competence and the confidence of the personnel and can lead to the timely treatment of allergic reactions in schoolchildren with FA, including the appropriate use of EIA.

The authors acknowledge the low number of participating schools, but the selection was random and stratified according to the different areas of the island. Despite this limitation, our results highlight, for the first time in Cyprus, the lack of compliance with legislation, and the inconsistencies among the different schools, the absence of standard protocols in schools for management of anaphylactic episodes, and inadequacy of appropriate training of the personnel, particularly in EIA use, but also regarding preventive measures for FA, such as sharing food and food labelling.

## 5. Conclusions

According to this study, the preparedness of primary school personnel in Cyprus did not meet safety standards regarding food-induced allergic reactions in schoolchildren with FA. The most frequently reported food allergens in schoolchildren were cereals, fruits, egg, chocolate and nuts, and most commonly recognized symptoms were wheals, itching, airway obstruction and wheezing. A gap in the knowledge about and the use of EIA in schools was revealed. There is a need for a written medical protocol to improve the management of allergic reactions by the school personnel, and training of primary school personnel in the recognition and management of FA reactions is recommended.

## Figures and Tables

**Table 1:** Questionnaire on food allergy for teachers.

Questionnaire on Food Allergy for Teachers
What is your position in the school? How many people work at this school? Do you know if any schoolchildren have presented a food allergic reaction during the past three years? If the previous answer is YES, how many children? Do you know in what food allergic reactions have occurred? Is there a school protocol regarding children with anamnesis of chronic medical conditions, like allergies? How are food-allergic children identified? Do you keep records of them? If the previous answer is YES, who is authorized to access in such information? Do you update the records? What type of data do the records include? Are you familiar with the symptoms of food allergy? If the previous answer is YES, can you list them? Are teachers and the rest of the personnel informed and trained to recognize food allergens and symptoms? Has an educational program been offered to them? What was the nature of the offered educational program and in how many sessions? What is the school protocol for the treatment of a severe anaphylactic reaction? What would your first action be? (A) contact the parents (B) remain to see how it goes (C) contact child’s family doctor (D) call emergency (E) administrate Anapen (since Anapen is the only available EIA in Cyprus, the brand name was used)? If you choose more than one of the above actions, can you place them in a row from the first to the last? Do you have Anapen available at school for severe food allergy reactions? If the previous answer is YES, where do you store it? What is your estimation on the percentage of teachers and non-teaching personnel that are familiar with Anapen’s availability? Does the school personnel know how to use Anapen? Is there a specially trained person to administrate Anapen in the case of anaphylaxis? Has the Cyprus Ministry of Education, Culture, Sport and Youth provided Directions on how to treat anaphylaxis or other food-related clinical features? Do you have any instructions on children to avoid sharing cutlery, glasses, home-prepared meals or snacks they buy from the school canteen? If YES, what is your estimation on the percentage of school personnel that know these instructions? Are children trained to follow such instructions? Do the parents know about that school instructions? Do you believe that school rules, forbiting to share things/foods, can negatively affect children’s personality and relationship? Is the school personnel informed on food labels and questionable ingredients? If the previous answer is YES, in what percentage? Do children get informed about food labelling as a part of their school education? Do you consider that you focus a lot on that health issue and thus resulting in a group of foods get excluded? Do you know any food allergy awareness organization? What is its name? Does the school get in contact with that organization? If the previous answer is YES, how much useful is? On a scale of 0 to 100, how much do you worry about the incidence with food allergies at school? If it near 100/or lower of 50, why? Do you give more or less sententiousness compared with other health problems? Do you think that this interview will motivate you to reconsider the risk of food allergy reactions? What type of actions would you propose specialists to offer at your school in order to raise awareness on food allergy?

## Data Availability

Data are available upon request to E.Vassilopoulou.
